# Supplementary material for: Genome-wide identification, characterization and gene expression of BES1 transcription factor family in grapevine (Vitis vinifera L.)
Source: Sci Rep. 2023 Jan 5;13:240. doi: 10.1038/s41598-022-24407-y (PMC9816167; doi:10.1038/s41598-022-24407-y)
Supplement: Supplementary file 3 — Supplementary Information. [file 41598_2022_24407_MOESM3_ESM.zip › Vvi_Atr/Vitis_vinifera.PN40024.v4.dna_sm.toplevel.fa.vs.Amborella_trichopoda.AMTR1.0.dna_sm.toplevel.fa.html/Atr-AmTr_v1.0_scaffold00227.html]

|  |  |  |  |  |  |  |  |  |  |  |  |  |  |
| --- | --- | --- | --- | --- | --- | --- | --- | --- | --- | --- | --- | --- | --- |
| Duplication depth | Reference chromosome | Collinear blocks | | | | | | | | | | | |
| 0 | Atr-ERM98953 |  |  |  |  |  |  |  |  |  |  |  |  |
| 0 | Atr-ERM98954 |  |  |  |  |  |  |  |  |  |  |  |  |
| 0 | Atr-ERM98955 |  |  |  |  |  |  |  |  |  |  |  |  |
| 0 | Atr-ERM98956 |  |  |  |  |  |  |  |  |  |  |  |  |
| 0 | Atr-ERM98957 |  |  |  |  |  |  |  |  |  |  |  |  |
